# Incidence of infectious diseases in infants fed follow-on formula containing synbiotics: an observational study

**DOI:** 10.1111/j.1651-2227.2010.01896.x

**Published:** 2010-11

**Authors:** Jean-Charles Picaud, Véronique Chapalain, Damien Paineau, Othar Zourabichvili, Francis RJ Bornet, Jean-François Duhamel

**Affiliations:** 1 Department of Neonatology, Croix-Rousse University Hospital, Claude Bernard University Lyon1Lyon, France; 2 Department of Methodology and Statistics, Quanta MedicalRueil-Malmaison, France; 3 Department of Methodology, Nutri-HealthRueil-Malmaison, France; 4 Department of Paediatrics, Caen University hospitalCaen, France

**Keywords:** Infections, Microflora, Nutrition, Probiotics, Probiotics

## Abstract

**Aim:**

Infectious diseases in infants are a major public health issue. Synbiotic-enriched formulas (EF) are intended to mimic the beneficial effects of human milk on infectious diseases. We performed an observational study in infants switching to follow-on formula to determine the effects of synbiotic-enriched formula compared to standard formula (SF).

**Methods:**

We recorded family characteristics, medical history and growth data, as well as the symptoms, severity and treatment of infectious diseases. Main outcome measures were compared after adjustments for baseline characteristics.

**Results:**

Between January and June 2007, 771 healthy infants were included in the study; 35.4% experienced at least one infectious disease during the 3-month study period. The most common were upper respiratory tract (24.1%), otitis (6.6%) and gastrointestinal infectious diseases (5.0%). Infants fed synbiotic-enriched formula had fewer infectious diseases overall (EF: 31.0%; SF: 40.6%; p = 0.005) and significantly fewer gastrointestinal infectious diseases (EF: 3.5%; SF: 6.8%; p = 0.03). During follow-up, weight gain was significantly higher (p = 0.0467) in infants fed synbiotic-enriched formula (18.3 ± 8.7 g/day) versus SF (16.9 ± 7.5 g/day).

**Conclusions:**

Supplementation with synbiotics may have beneficial effects on the incidence of infectious disease and growth in infants. Further studies are needed determine optimal doses and composition of synbiotics in infant formula.

## Introduction

In the United States, more than 40% of all infant hospitalizations are attributable to infectious disease, resulting in total annual hospital costs of approximately $690 million in 2003 (1). Diarrhoeal diseases and digestive tract infections are the most common infectious diseases in infants. Rotavirus gastroenteritis, for example, is known to affect nearly every child by age 5 (2). Infectious diseases are very frequent and are associated with substantial costs. Preventive strategies could include functional nutrition of infants.

Special attention should be paid to infectious disease incidence in infants switching from exclusive breast feeding to follow-on formula. Breast feeding has been shown to have a number of beneficial effects in infants, including protection against infectious and allergic diseases. Those effects are probably mediated in part through modulation of intestinal microflora (3,4). Oligosaccharides are likely to be involved in this beneficial modulation of microflora in breast-fed infants (5), because they influence gastrointestinal development and reduce respiratory and gastrointestinal illness in infants (6).

To mimic these beneficial effects of human milk on gut microflora, prebiotic- and probiotic-enriched formulas have been developed in the past 10 years. It has been suggested that these formulas could reduce the incidence of infectious diseases and atopy (7–9). Recently, new formulas have been proposed that contain synbiotics, i.e. combined prebiotics and probiotics (10,11). Synbiotics are expected to be more beneficial than probiotics or prebiotics alone, owing to synergistic effects.

In this study, we investigated the incidence and types of infectious diseases in healthy infants during the first three months of consumption of a new symbiotic-enriched follow-on formula (EF) compared to infants consuming standard formula (SF).

## Patients and Methods

### Study design

A prospective, multicentre, open study with two intervention groups was carried out in France, with the collaboration of family paediatricians. Three-hundred paediatricians were randomly selected from the 1200 paediatricians practicing in France and were invited by letter to participate in the study; 166 accepted. Infants aged between 4 and 6 months of age who needed follow-on formula were enroled by 166 paediatricians scattered all over the French territory, after informed written consent was obtained from parents. Inclusion criteria were birth weight between 2 and 4.5 kg and no history of neonatal or digestive disorders. Infants who had an immune deficiency were excluded. Because of the pilot nature of the study and in line with the proposal by participating family paediatricians, an open study design was applied. This study was registered with the French Medical Council to the National Consultative Committee on Data Processing in Biomedical Research.

### Study protocol

During the inclusion visit, paediatricians alternatively prescribed use of either one of the standard follow-on formula (SF) or a synbiotic-enriched formula (EF) for 3 months. As recommended in routine practice, follow-on formula was proposed by paediatricians at the time of diversification, i.e. at beginning of diversification or just before diversification. Both SF and EF formulas were commercially available in France. SF was let to the choice of the paediatrician who chooses one of the commercially available follow-on formulas without probiotics and prebiotics. The nutrient composition of SF corresponded to values determined by European rules (energy: 70–74 kcal/dL, protein: 1.8–1.9 g/dL). The only EF available in France at the time of the study was Nutriben 2 Prebiotiques and Probiotiques (Nutriben, Les Ulis, France). Its composition corresponded also to values determined by European rules (energy: 74 kcal/dL, protein: 1.9 g/dL). This formula contains fructooligosaccharides (28 mg/g of powder) and two probiotic strains (*Bifidobacterium longum* at 10^7^ UFC/g of powder and *Streptococcus thermophilus* at 10^6^ UFC/g of powder). Neither EF nor SF contained nucleotides or other immunological factors.

### Study end points and data collection

Primary outcome was the incidence of infectious diseases. All episodes of infections were recorded during a follow-up of 3 months. The diagnosis was made by the family paediatrician based on specific symptoms, and they were given instructions on how to collect information about infectious diseases on the basis of standardized definitions. Acute diarrhoea was defined as a stool pattern with 3 or more loose or watery stools per day lasting for at least 3 days. Pharyngitis, laryngitis and tracheitis were considered as upper respiratory tract infections. Antibiotic courses were also recorded.

At inclusion, paediatricians collected data about the infant’s birth, family history, parents’ smoking behaviour, frequency of infections in parents, day care situation (day care centre or home care), medical history (allergies and number of infectious diseases since birth, treatments and vaccines), feeding practices since birth and clinical examination data. At each monthly follow-up visit, they collected anthropometric data, information about the occurrence of infectious diseases since the last visit and at follow-up visits, treatments and vaccination at follow-up visit and changes in feeding since last visit (such as type of formula, amount of formula, etc.). Secondary outcomes measured were weight and length gain.

The survey protocol and questionnaires were presented in person to all participating physicians. Telephone and email helplines were set up at the beginning of the study and maintained throughout for paediatricians and parents who sought assistance or advice on any topic related to the study. Quality audits were performed on six randomly selected investigators by clinical research assistants to verify that physicians were compliant with the protocol.

A questionnaire was developed for parents to record suspected infectious diseases during the 3-month study period. Prior to the start of the study, parents were given instructions on how to use the questionnaire and how to contact the dedicated study hotline if necessary. Every time an infectious disease was suspected, parents completed items regarding the symptoms. If relevant, they also recorded additional information about day care centre absences, hospitalization, diagnosis and treatment.

Paediatricians read the parent questionnaire and questioned parents at each visit about infectious diseases noted by parents. The final visit was scheduled to take place 3 months after inclusion. Before statistical analysis, data collected from the parent questionnaire and data collected by the paediatrician were compared for each infant. Infectious diseases were categorized as ‘possible’ or ‘confirmed.’ Only infectious diseases reported to be ‘confirmed’ by paediatricians were included in the analysis.

### Statistical analysis

Infectious disease incidence during the 3 months of formula consumption was the primary outcome measure. Incidence was expressed as the proportion of babies with one or more episodes of certain infectious diseases. Based on data published previously (12), we hypothesized that using milk formula supplemented with synbiotics would reduce the incidence from 40% to 30%. We calculated that for a power of 80% (β = 0.20) and at a significance level of .05 (α = 0.05), we needed 376 infants in each feeding group to detect a 10% difference between the EF and SF groups. To allow for an expected 10% dropout rate, we decided to recruit 836 infants (418 infants in each group).

Continuous variables were expressed as mean ± standard deviation. Exploratory intergroup comparisons were performed according to the type of follow-on formula consumed (SF or EF). All tests were two-sided. The risk of type 1 error (α) was set at .05 for the entire study. Means were compared using Student’s *t*-tests. Categorical variables were compared using χ^2^ or Fisher’s exact tests as appropriate. For comparison of main outcome measures, adjustments of baseline characteristics were performed using logistic regression models (infectious disease incidence) or covariance analysis (mean number of infectious diseases during follow-up). There was no approximation of missing data; instead, missing data items were omitted from the statistical tests and not taken into account when calculating percentages. The following were considered to be deviations from the protocol: change of follow-on formula during study period (so that no follow-on formula was consumed at least 75% of the follow-up time) and no data for the main outcome measure (infectious disease incidence). Intention-to-treat (ITT) as well as per-protocol (PP) analyses were performed.

To identify potential prognostic factors for infectious disease in 4- to 6-month-old infants, a stepwise logistic regression multivariate analysis was performed in the ITT population in an exploratory way with the ‘Occurrence of infectious disease (Yes/No)’ as the dependent factor. Variables considered for the multivariate modelling were variables with p < 0.20 in univariate logistic regressions (data not shown). The entry and removal criteria for stepwise selection were set to 0.10 and 0.15, respectively. The deviance and Pearson goodness-of-fit statistics as well as the Hosmer–Lemeshow goodness-of-fit were calculated. All variables included in the model were tested for collinearity. The 95% confidence interval (95%CI) for the odds ratio (OR) was calculated. Data analysis was performed using the SAS statistical program, version 8.2 (SAS Institute, Inc., Cary, NC, USA).

## Results

### Participants

Between January 2007 and June 2007, 824 infants were eligible to participate in the study (Fig. S1). Fifty-three infants were not included because parents refused to provide permission. Of the baseline sample, 94% (771 infants) were included in the study: 422 consumed synbiotic-enriched formula (EF group) and 349 consumed standard formula (SF group). Most infants (58%) were included before the 1st of April, and there was no significant difference between feeding groups (EF: 60.6%, SF 54.9%, p = 0.109). Baseline characteristics are presented in Table 1. At birth, there was no difference between feeding groups for body weight (EF: 3310 ± 410 g vs SF: 3270 ± 510 g, p = 0.559) and for length (EF: 49.6 ± 2.0 g vs SF: 49.7 ± 2.1 g, p = 0.647). Half (50.2%) of the subjects were male patients. The characteristics of the 53 non-included subjects were similar to those of included subjects (data not shown). Of the 771 subjects, 27 subjects in the EF group and 52 subjects in the SF group presented with at least one major protocol deviation and were excluded from the PP population. Characteristics of these excluded subjects were similar (data not shown). PP analysis was thus performed on 692 infants (84% of the baseline sample) (Figure S1). PP results are not shown but were in line with results for the ITT population (presented later).

**Table 1:** Baseline characteristics in the intention-to-treat group (Mean ± standard deviation)

	EF group	SF group	All infants
			
n	422	349	771
Age at enrolment (day)	153 ± 44	151 ± 34	152 ± 40
Weight at enrolment (kg)	6.96 ± 1.02	6.98 ± 0.90	6.97 ± 0.96
Length at enrolment (cm)	64.0 ± 3.7	64.2 ± 3.8	64.1 ± 3.81
Family history of allergy (%)	38.4*	27.0	33.2
Personal history of allergy (%)	16.1*	10.0	13.3
Number of siblings (n)	0.69 ± 0.84	0.60 ± 0.85	0.66 ± 0.84
Frequent infectious episodes in parents (%)	7.7	5.2	6.5
Smoking in the family home (%)	15.4	17.9	16.5
Number of infectious diseases since birth (n)	1.0 ± 1.3*	0.7 ± 1.2	0.9 ± 1.2
Partly or exclusively breast feeding at inclusion (%)	47.2	45.5	46.4
Consumption of prebiotic- or probiotic-enriched infant formula before inclusion (%)	20.6	15.4	18.3
Dietary diversification before inclusion (%)	85.0	87.4	86.1
Daily volume of follow-on formula prescribed (mL)	748 ± 180	752 ± 176	750 ± 178
Addition of wheat to follow-on formula (%)	27.8	28.4	28.1

* p < 0.05 versus SF (Student's *t*-test or χ^2^/Fisher's exact test).

### Feeding practices and duration of follow-up

Before changing for a follow-on formula (inclusion), infants were exclusively breast-fed (22%), exclusively formula fed (54%) or received human milk together with formula (24%). The proportion of subjects partly or exclusively breast-fed at inclusion was similar in both groups (Table 1). The mean daily volume intake was 750 ± 178 mL/day at the day before inclusion; this decreased to 629 ± 183 mL/day at the end of study period. It was similar between groups both at inclusion (p = 0.68) and at the final visit (p = 0.87). Infant cereals were consumed by 28.1% of the cohort, and similar proportions of infants consumed cereals in both groups (p = 0.86). The mean follow-up duration was 2.8 ± 1.0 months and was identical between groups (p = 0.65).

### Growth

During the study period, weight gain was significantly higher in infants fed EF (18.3 ± 8.7 g/day) than in those fed SF (16.9 ± 7.5 g/day) (p = 0.047). The increase in length tended to be also greater in the EF group (0.47 ± 0.22 cm/week) than in the SF group (0.45 ± 0.25 cm/week), but the difference was not statistically significant (p = 0.069).

### Incidence of infectious diseases

In the ITT analysis, 35.4% of all infants experienced at least one infectious disease during follow-up (Table 2). Upper respiratory tract infections, occurring in almost one out of four infants, were the most common type of infectious disease. The incidence of infectious disease was significantly lower in the EF group than in the SF group (p = 0.005), even after adjustment by logistic regression to take into account the number of infectious diseases since birth (p = 0.0004) and the personal (p = 0.0031) or family (p = 0.0015) history of atopy. No difference was found between groups when considering only the incidence of infectious diseases during the first month of follow-up (EF: 16.8%, SF: 18.3%, p = 0.58). Among subjects born after the 1st of April, there was significantly less infections in EF group (29,1%) than in SF group (40.1%) (p = 0.037). Among subjects born before the 1st of April, there was a tendency for less infections in EF group (32.7%) than in SF group (41.4%) (p = 0.06). The mean number of infectious diseases per month tended to be lower in the EF group than in the SF group (p = 0.07), even after adjustment by covariance analysis (p = 0.08). Regarding the types of infectious diseases, only the incidence of gastrointestinal infections differed between groups (p = 0.03); accordingly, fewer infants in the EF group were prescribed anti-diarrhoea follow-on formula (EF: 3.4%; SF: 7.4%; p = 0.02). There was a tendency towards fewer antibiotic and antipyretic prescriptions in the EF group compared to the SF group (Table S1).

**Table 2:** Infectious disease incidence and type during follow-up in the intention-to-treat group (Mean [CI95])

	EF group	SF group	All infants
			
n	419	347	771
Infectious disease†(%)	31.0*	40.6	35.4
Upper respiratory tract infections (%)	22.0	26.6	24.1
Otitis (%)	5.2	8.3	6.6
Acute diarrhoea (%)	3.5*	6.8	5.0
Lower respiratory tract infectious disease (%)	5.2	4.3	5.8
Genital and urinary infectious disease (%)	0.2	0.2	0.2
Dermatologic infectious disease (%)	0.7	1.1	0.9
Ophthalmologic infectious disease (%)	1.6	1.4	1.5
Virus-related dermatologic infectious disease (%)	0.9	1.4	1.1
Monthly number of infectious diseases (n)	0.23 [0.18; 0.27]	0.25[0.21; 0.30]	0.24[0.21; 0.27]

* p < 0.05 versus SF (Student’s *t*-test or χ^2^/Fisher's exact test).

† Percent of infants with at least one infectious disease during follow-up.

Multivariate stepwise logistic regression revealed that the following three factors were independently associated with an increased risk of infectious disease in infants: frequent infections in parents, the consumption of SF rather than EF and the occurrence of infections before inclusion (Table 3). The absence of breast feeding at inclusion tended to be associated. The final model included the 668 subjects with no missing data in any of the four independent variables of the model. The goodness-of-fit of the model was quite good (Hosmer–Lemeshow: Chi-square = 4.41, p = 0.73; Deviance and Pearson criteria: Chi-square = ∼1).

**Table 3:** Multivariate stepwise logistic regression analysis (n = 668) with the occurrence of infectious disease as the independent factor.

Parameter	Odds ratio	95%CI	p*
Frequent infection in parents	2.797	[1.462; 5.349]	0.0019
Standard follow-on formula as feeding regimen	1.926	[1.380; 2.689]	0.0001
Infection in infants before inclusion	1.883	[1.338; 2.649]	0.0003
Absence of breast feeding at inclusion	1.384	[0.992; 1.930]	0.0558

* Wald test.

## Discussion

In this clinical study, healthy 5-month-old infants fed EF had a reduced incidence of infectious disease compared to infants fed SF. To our knowledge, this is the first longitudinal study investigating the incidence of infectious diseases in infants switching to follow-on formula EF.

The strengths of this study were the relatively large sample size, the detailed follow-up of infectious diseases and nationwide recruitment. The study was initially designed to be conducted solely during winter months (November–April) to investigate the effects of synbiotics during the season with the highest incidence of infectious diseases. However, because of recruitment delays, it was not possible to start the study before January, and the last patients were included in June. This shift in the study period may have resulted in underestimation of the clinical effects of synbiotics on infants. Intergroup comparisons are still relevant, because similar percentages of infants were included before April (i.e. during winter) in the two groups.

The two feeding groups were unbalanced. It could have been partly related with the delay in the study set-up. Even though our target number of 418 subjects was not achieved in the SF group, the inclusion time period was not extended beyond June, because summer is a period of low risk for infections. However, the number of subjects included in the two groups was sufficient to observe a significant difference in the main outcome criteria. It could have been also related to an inclusion bias as paediatricians included more infants in the EF group. They could have been influenced by the familial and personal history of atopy or the greater number of infections since birth, as paediatricians had probably previously heard about the potential positive effect on health of prebiotics or probiotics. It could induce an overestimation of synbiotic effects on prevention of infections in this specific population. However, the difference in prevalence of infectious diseases between the two feeding groups persisted after adjustment for the difference in the number of infections since birth and for difference in familial or personal history of allergy.

In our study, around one-third of infants had at least one infectious disease during the 3-month follow-up period. We only collected clinical data for infectious diseases, but we compared information collected by paediatricians with the information provided by parents. Only confirmed infectious diseases were used in our analyses. Our results were in line with previous studies, indicating that the present study probably did not overestimate the incidence of infectious diseases. Recently, a large Australian cohort study reported that half of all children were hospitalized at least once by age two; infections (mainly respiratory and gastrointestinal) were the most common reason for hospitalization, accounting for 34% of all admissions (13). In a double-blind, randomized, placebo-controlled trial of healthy children aged 1–6 years attending day care centres, conducted over 7 months in Finland in 2001, the incidence of respiratory tract infections (lower and upper) was 39% in children consuming formula supplemented with *Lactobacillus* GG and 47% in children consuming standard formula (12). Another study reported a high incidence of upper respiratory tract infections with frequent complications, such as otitis and sinusitis: in children aged 6–35 months, 30% of upper respiratory tract infections were complicated by acute otitis media (14). Respiratory tract infections are associated with parental smoking; however, in the present study, only 15.4% of families reported parental smoking, and there was no difference in parental smoking in EF group and in the SF group. It is likely that frequent exposure to infectious agents was responsible for the high incidence of respiratory tract infections; this is supported by epidemiological observations in France at the time of the study.

The most convincing finding is the preventive effect exerted against intestinal infections. This was supported by the reduction in the incidence of diarrhoeal episodes in infants receiving the symbiotic-enriched formula. It is noteworthy that probiotics (15) and prebiotics (9) when given separately have been found to give protection against acute gastroenteritis. Because our study was not randomized, the difference in infections between the two groups could have been observed by chance. For example, the number of infectious diseases since birth was significantly higher in the EF group than in the SF group. However, our results persisted after adjustment for that difference between the two groups.

EF was associated with a lower incidence of infectious disease in our paediatric cohort compared to SF, both in ITT analysis and also in PP analysis (PP: EF: 30.1%; SF: 39.0%; p = 0.001 after adjustment). This was in line with previous studies, which reported that prebiotics and probiotics had beneficial effects (7–9). In children over 2 years of age, a synbiotic preparation was found to effectively treat atopic dermatitis (16). Another study in children with short bowel syndrome showed that synbiotic therapy improves systemic immunonutritional status (11), and there was a case report that a novel synbiotic therapy improved the intestinal function of a paediatric patient with a laryngotracheoesophageal cleft (17). In acutely ill children aged 1–6 years, oral supplements containing synbiotics increased energy intake and promoted weight gain (18). Regarding intergroup comparisons of EF versus SF, this open study was intended to be a preliminary analysis: further randomized, double-blind, controlled trials are needed to confirm the clinical benefits of EF.

The mechanisms of action of synbiotics are probably similar to those of prebiotics and probiotics, with possible synergistic effects as demonstrated in animals (19). The mechanisms include resistance to colonization by pathogens through production of inhibitory substances, blockage of adhesion sites, competition for nutrients, degradation of toxin receptors and stimulation of immunity (20).

The preventive effect on infections was not the only clinical effect we observed. Infants fed the EF gained significantly more weight than those fed SF, but that difference is not clinically relevant over such a short period. It is always possible that this result was non-specific; however, weight gain was observed in parallel with increases in length. Such a difference could be more important over a longer period. This positive effect on growth could be related to the reduced incidence of infectious diseases during this period or could alternatively be attributable to the effect of probiotics or prebiotics on digestive microflora (15). A positive relationship between digestive microflora and growth has been suggested in animals (21,22), preterm infants (23) and older infants treated with antibiotics (18). Further investigators should be encouraged to evaluate specifically the growth in infants fed with formulas that are able to have an impact on intestinal microflora.

In conclusion, this clinical study indicates that infectious diseases affect more than one-third of healthy 5-month-old infants in the 3 months following a switch to follow-on formula and suggests an association between the administration of a synbiotic-enriched follow-on formula and clinical effects in healthy infants. Randomized clinical trials are needed to understand the mechanisms and clinical effects of synbiotics, especially in infants less than 1 year of age. The optimal daily doses and formulations of prebiotics and probiotics must also be established.

## Abbreviations

EF: synbiotic-enriched formula
ITT: intention-to-treat
PP: per-protocol
SF: standard formula

## SUPPORTING INFORMATION

**Figure S1** Study design and participant flow.

**Table S1** Medical treatments and vaccine administration during follow-up in the intention-to-treat group.
