# Supplementary material for: Incidence of infectious diseases in infants fed follow-on formula containing synbiotics: an observational study
Source: Acta Paediatr. 2010 Nov;99(11):1695–700. doi: 10.1111/j.1651-2227.2010.01896.x (PMC3034191; doi:10.1111/j.1651-2227.2010.01896.x)
Supplement: Supplementary file 1 [file apa0099-1695-SD1.doc]

**Figure 1.**

***ITT analysis***

***PP analysis***

53 refusals

Allocated to the EF group: 422

Allocated to the SF group: 349

**824 eligible infants**

771 infants included

Deviations from protocol

27 infants

52 infants

**395 infants fed EF**

**297 infants fed SF**
