# Supplementary material for: Incidence of infectious diseases in infants fed follow-on formula containing synbiotics: an observational study
Source: Acta Paediatr. 2010 Nov;99(11):1695–700. doi: 10.1111/j.1651-2227.2010.01896.x (PMC3034191; doi:10.1111/j.1651-2227.2010.01896.x)
Supplement: Supplementary file 2 [file apa0099-1695-SD2.doc]

**Table 3.** Medical treatments and vaccine administration during follow-up in the intention-to-treat group

|  | EF group | SF group | All infants |
| --- | --- | --- | --- |
| n | 419 | 347 | 771 |
| Antibiotic prescription (%) | 8.0 | 11.7 | 9.7 |
| Antipyretic prescription (%) | 34.1 | 39.8 | 36.7 |
| Other medical treatment (%) | 22.7 | 26.9 | 24.6 |
| Vaccine administration (%) | 77.0 | 79.3 | 78.0 |

No significant difference
